# A study of factors impacting disease based on the Charlson Comorbidity Index in UK Biobank

**DOI:** 10.3389/fpubh.2022.1050129

**Published:** 2023-01-09

**Authors:** Changcong Wang, Xinyue Zhang, Bai Li, Dongmei Mu

**Affiliations:** ^1^Division of Clinical Research, The First Hospital of Jilin University, Changchun, China; ^2^Department of Medical Informatics, School of Public Health, Jilin University, Changchun, China; ^3^Department of Colorectal and Anal Surgery, General Surgery Center, First Hospital of Jilin University, Changchun, China

**Keywords:** multimorbidity, Charlson Comorbidity Index (CCI), deprivation indices, impact factors, Restricted Cubic Spline (RCS)

## Abstract

**Objective:**

With advances in medical diagnosis, more people are diagnosed with more than one disease. The damage caused by different diseases varies, so relying solely on the number of diseases to represent multimorbidity is limited. The Charlson comorbidity index (CCI) is widely used to measure multimorbidity and has been validated in various studies. However, CCI's demographic and behavioral risk factors still need more exploration.

**Methods:**

We conduct multivariate logistic regression analysis and restricted cubic splines to examine the influence factors of CCI and the relationship between covariates and risk of CCI, respectively. Our research employs the Multivariate Imputation by Chained Equations method to interpolate missing values. In addition, the CCI score for each participant is calculated based on the inpatient's condition using the International Classification of Diseases, edition 10 (ICD10). Considering the differences in the disease burden between males and females, the research was finally subgroup analyzed by sex.

**Results:**

This study includes 5,02,411 participants (2,29,086 female) with CCI scores ranging from 0 to 98. All covariates differed between CCI groups. High waist-hip ratio (WHR) increases the risk of CCI in both males [OR = 19.439, 95% CI = (16.261, 23.241)] and females [OR = 12.575, 95% CI = (11.005, 14.370)], and the effect of WHR on CCI is more significant in males. Associations between age, Body Mass Index (BMI) and WHR, and CCI risk are J-shaped for all participants, males, and females. Concerning the association between Townsend deprivation index (TDI) and CCI risk, the U-shape was found in all participants and males and varied to a greater extent in males, but it is a J-shape in females.

**Conclusions:**

Increased WHR, BMI, and TDI are significant predictors of poor health, and WHR showed a greater role. The impact of deprivation indices on health showed differences by sex. Socio-economic factors, such as income and TDI, are associated with CCI. The association of social status differences caused by these socioeconomic factors with health conditions should be considered. Factors might interact with each other; therefore, a comprehensive, rational, and robust intervention will be necessary for health.

## 1. Introduction

Communicable and non-communicable diseases have always been significant problems affecting human health and quality of life, especially for middle-aged and elderly people. In 2019, ischaemic heart disease and stroke were already significant causes of disability-adjusted life years (DALYs) for people aged above 25 years. They ranked as the top two DALYs for people aged above 50 years (1). The Global Burden of Disease Study 2016 reported that cardiovascular disease was the non-communicable disease responsible for the highest number of secondary deaths, followed by neoplasms and chronic respiratory diseases (2). With advances in medical diagnosis, more people have been found to suffer from two or more major diseases, known as comorbidity or multimorbidity. Although “comorbidity” and “multimorbidity” (3) were defined as different Medical Subject Headings (MeSH) terms in 2018, both focus on the occurrence of multiple chronic conditions in the same person. In contrast, “multimorbidity” preferred that no one disease had priority in the case of coexisting diseases (3). Whether it was “comorbidity” or “multimorbidity,” the co-existence of multiple conditions was already a complex issue because multiple co-existing diseases might interact with each other, and there could be complex interactions and potential associations (4).

A Scotland study identified that the number of diseases and the proportion of people with multimorbidity increased with age, and almost all people over 65 had at least one disease (5). Not only was the healthy lifespan of older people negatively affected by multimorbidity (6), but the Australian cohort study also found that the coexistence of multiple conditions was becoming increasingly common at younger ages (7). In Europe, multimorbidity lowered the quality of life and raised the costs of medication, health care, etc., i.e., the cost of health (8, 9). There are limitations in relying solely on the number of diseases to represent multimorbidity; for example, the degree of damage caused by different diseases varies. Weighted measures focusing on co-morbidities have provided better predictions than assessing individual diseases alone (10). We, therefore, use the Charlson Comorbidity Index (CCI) to represent individual multimorbidity (11).

The CCI is a widely used measure of multimorbidity and has been validated in various studies (11, 12). Several studies have demonstrated that CCI accurately predicted many types of patients, including cancer patients and those in intensive care (13–15). The efficient and effective management of multimorbidity has become a new task and challenge for patients and professionals in the field of public health. Risk factors for many diseases, including obesity (16) and smoking (17) have been identified. However, CCI's demographic and behavioral risk factors were not explored much. Therefore, this study aimed to explore the possible influencing factors associated with CCI using the United Kingdom (UK) biobank dataset.

## 2. Materials and methods

### 2.1. Population and study design

Our study is a cross-sectional analysis based on the UK Biobank (Application Title: Integration of clinical data and genomic data to construct diagnosis and prognosis system for digestive diseases and related complications, Application ID: 84347). The UK Biobank is the world's most detailed, long-term prospective health study. Recruitment occurred in 22 centers in Scotland, England and Wales between 2006 and 2010. People aged 40–69 living in the UK were invited by mail inquiry and telephone to their nearest assessment center, where trained professionals collected baseline information, physical measures and biological samples. Of the 9.23 million people invited to join the UK Biobank, 5,03,317 (5.45%) agreed and were recruited (18). The UK Biobank assessment process had five components which include: written consent, touch screen questionnaires, face-to-face interviews, measurements and blood, urine and saliva sample collection. All participants approved this UK Biobank study and provided written informed consent to participate in the UK Biobank study. Further details of these measurements, study design, and data collection are available in the UK Biobank online protocol and study protocol (http://www.ukbiobank.ac.uk). We obtained data on 5,02,411 participants from UK Biobank when our application was approved. For this study, we included all participants and conducted both the primary and sensitivity analyzes.

### 2.2. Charlson comorbidity index

The Charlson Comorbidity Index (CCI) is calculated based on the inpatient's disease obtained from the diagnosis made during the admission (Supplementary Table 1). There are 17 categories of conditions that could contribute to the CCI score, each assigned a value ranging from 1 to 6 depending on the condition (11). We assess CCI scores using the International Classification of Diseases, edition 10 (ICD-10). Each disease category in the CCI corresponds to one or more ICD-10 codes. We assign each ICD-10 code to a corresponding disease weighting score based on Quan et al. (19). The total score for the CCI is a simple sum of the weights, and higher scores represent more severe comorbidity or multimorbidity.

### 2.3. Ascertainment of covariates

Townsend deprivation index (TDI) (20), which combines information on housing, employment, and car availability, was calculated based on census and postcode prior to participant recruitment. The index mainly measures socioeconomic status, with higher values meaning higher deprivation.

Body Mass Index (BMI) was calculated as weight (kg)/height (m)^2^. Weight was measured using a Tanita BC-418 MA body composition analyzer, accurate to 0.1 kg, and height was measured using a Seca 202 height measure. Participants were required to remove their shoes and heavy clothing while the measurements were being taken. Waist-hip ratio (WHR) was calculated as waist circumference/hip circumference. Hip and waist circumference (at the level of the umbilicus) will be measured using a Wessex non-stretchable sprung tape measure and entered manually by staff. Trained staff carried out these measurements (21).

In addition, we also select age, ethnicity (22), income, International Physical Activity Questionnaire (IPAQ) (23), smoking, alcohol, maternal smoking around birth, illnesses of father, illnesses of mother and illnesses of siblings and as covariates for the study. These illnesses include mainly Prostate cancer (males only), Severe depression, Parkinson's disease, Alzheimer's disease/dementia, Diabetes, High blood pressure, Chronic bronchitis/emphysema, Breast cancer (females only), Bowel cancer, Lung cancer, Stroke, and Heart disease. Data on sociodemographics, income, IPAQ, smoking, alcohol, maternal smoking around birth and relatives' illnesses were collected from the touch screen questionnaire. Information on CCI and all covariates were obtained from baseline characteristics in 2006–2010.

### 2.4. Statistical analysis

In the primary analysis, all participants are included in the study. Responses that were “Preferred not to answer,” “uncertain/unknown,” or invalid are recoded as missing or null. Missing values are interpolated using the Multivariate Imputation by Chained Equations method with the R software “mice” package (with 5 imputed datasets, 10 iterations, and random forest method) and divide participants into two groups according to whether CCI = 0. CCI = 0, and CCI > 0 indicated good and poor health conditions, respectively. Continuous variables with normal distribution are described using mean ± standard deviation ($\bar{x}$ ± sd) and Student's *t*-test for comparison between groups; continuous variables with non-normal distribution are described using median and quartiles [M (Q1, Q3)] and Mann-Whitney *U*-test for comparison between groups; categorical variables are described using frequencies and percentages, and χ^2^ test is used for the analysis of differences in distribution.

We conduct a univariate logistic regression analysis with the CCI group as the dependent variable. Then the significant independent variables (*P* < 0.05) are included in a multivariate logistic regression analysis to explore possible impact factors. Discriminatory is assessed based on the mean of the area under the curve (AUC) for 10-fold cross-validation. We also perform Restricted Cubic Spline (RCS) curves to model the association of age, TDI, WHR, and BMI with CCI risk while adjusting for general condition variables such as ethnicity, smoking, alcohol, income, and maternal smoking at birth. Considering the differences in the burden of disease between males and females (24), the research is finally subgroup analyzed by sex.

For a sensitivity analysis of the preliminary study, we repeat the analyzes, excluding cases with missing values, to compare whether there is a significant change in the primary outcome. Using R software version 4.1.2 for all data analyzes in this research.

## 3. Results

**Figure 1:**
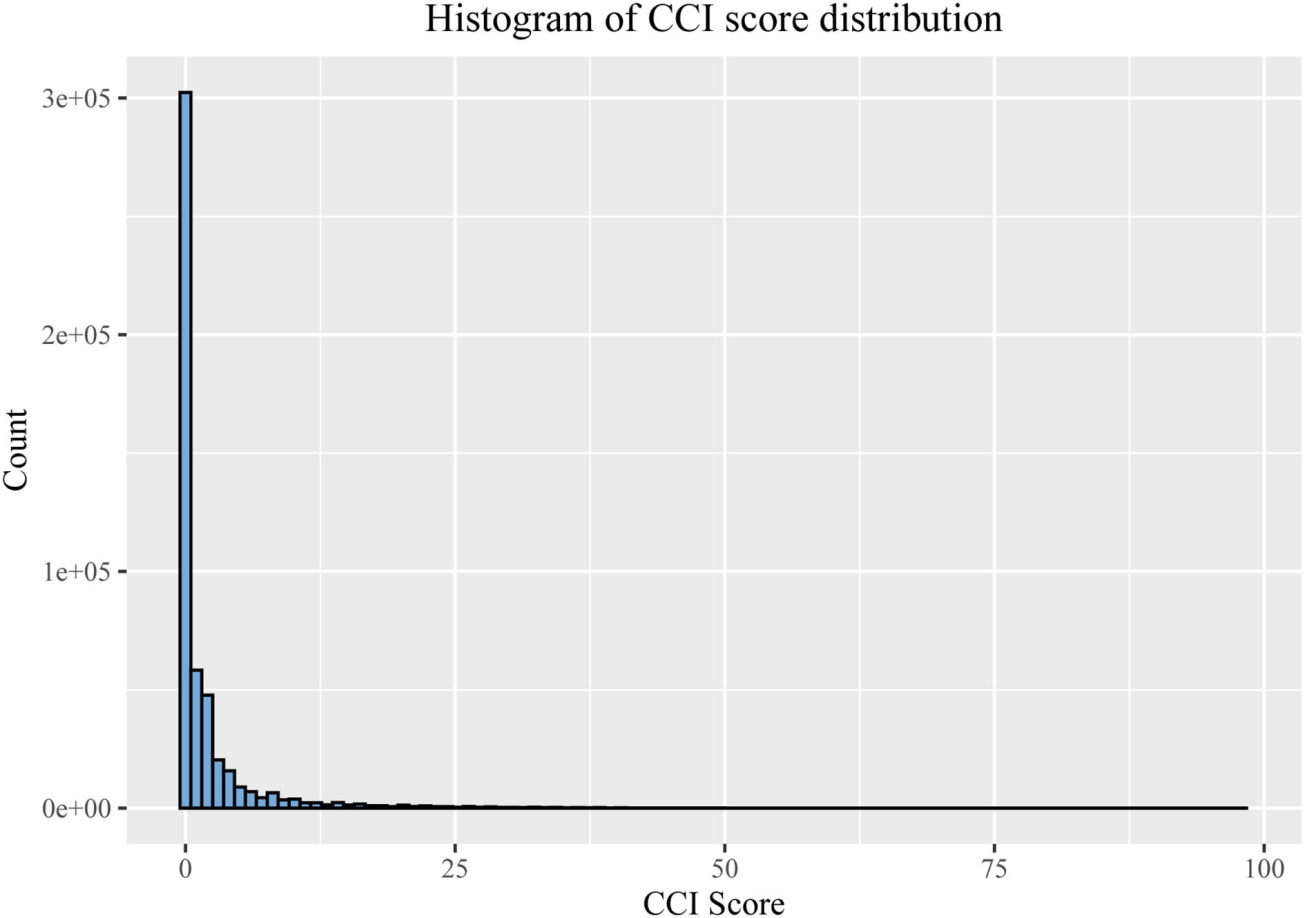
Distribution of different CCI scores.

UK Biobank investigators sent postal invitations to 92,38,453 people and 5,03,317 participants agreed to join the study cohort, for a participation rate of 5.45%. We ultimately obtain 5,02,411 participants from the UK Biobank application and include all of them in this study, with 2,29,086 males (aged: 37–73) and 2,73,325 females (aged: 39–71) (Table 1). CCI score equals the sum of individual scores, and the range is 0–98 points (males: 0–98; females: 0–93) (Figure 1). Following the AUROC (area under the receiver operating characteristic curve), our multivariate logistic models all possessed robust discrimination (AUROC means for all participants, males and females, were 0.707, 0.723, and 0.688, respectively, Supplementary Figure 1).

**Table 1:** General characteristics of the CCI group [Median (Q1, Q3)/*n* (%)].

**Variables**	**Total** **(*n* = 5,02,411)**	**CCI = 0** **(*n* = 3,02,344)**	**CCI > 0 (*n* = 2,00,067)**	***P*** **-value**
**Sex**				
Female	2,73,325 (54.4)	1,72,451 (57)	1,00,874 (50.4)	<0.001
Male	2,29,086 (45.6)	1,29,893 (43)	99,193 (49.6)	
Age (years)	58 (50, 63)	55 (48, 61)	61 (54, 65)	<0.001
**Ethnic**				
Asian or Asian British	11,553 (2.3)	6,642 (2.2)	4,911 (2.5)	<0.001
Black or Black British	8,131 (1.6)	4,803 (1.6)	3,328 (1.7)	
Mixed	2,969 (0.6)	1,875 (0.6)	1,094 (0.5)	
Others	4,596 (0.9)	2,817 (0.9)	1,779 (0.9)	
White	4,75,162 (94.6)	2,86,207 (94.7)	1,88,955 (94.4)	
BMI (Kg/m^2^)	26.75 (24.14, 29.91)	26.22 (23.77, 29.15)	27.62 (24.82, 31.08)	<0.001
WHR	0.87 (0.8, 0.94)	0.86 (0.79, 0.92)	0.9 (0.83, 0.96)	<0.001
**Income (£)** ^#^				
<18,000	1,18,903 (23.7)	54,889 (18.2)	64,014 (32)	<0.001
18,000–31,000	1,28,975 (25.7)	73,154 (24.2)	55,821 (27.9)	
31,000–52,000	1,29,124 (25.7)	83,964 (27.8)	45,160 (22.6)	
52,000–1,00,000	99,117 (19.7)	70,546 (23.3)	28,571 (14.3)	
>1,00,000	26,292 (5.2)	19,791 (6.5)	6,501 (3.2)	
TDI	−2.14 (−3.64, 0.55)	−2.27 (−3.71, 0.23)	−1.9 (−3.52, 1.04)	<0.001
**IPAQ**				
Low	95,267 (19)	52,824 (17.5)	42,443 (21.2)	<0.001
Moderate	2,05,023 (40.8)	1,24,503 (41.2)	80,520 (40.2)	
High	2,02,121 (40.2)	1,25,017 (41.3)	77,104 (38.5)	
**Smoking**				
Never	2,75,033 (54.7)	1,79,058 (59.2)	95,975 (48)	<0.001
Previous	1,74,038 (34.6)	95,728 (31.7)	78,310 (39.1)	
Current	53,340 (10.6)	27,558 (9.1)	25,782 (12.9)	
**Alcohol**				
Never	22,492 (4.5)	11,917 (3.9)	10,575 (5.3)	<0.001
Previous	18,174 (3.6)	8,273 (2.7)	9,901 (4.9)	
Current	4,61,745 (91.9)	2,82,154 (93.3)	1,79,591 (89.8)	
**Maternal smoking around birth**				
No	3,54,668 (70.6)	2,16,411 (71.6)	1,38,257 (69.1)	<0.001
Yes	1,47,743 (29.4)	85,933 (28.4)	61,810 (30.9)	
**Illnesses of father**				
No	1,16,383 (23.2)	74,046 (24.5)	42,337 (21.2)	<0.001
Yes	3,86,028 (76.8)	2,28,298 (75.5)	1,57,730 (78.8)	
**Illnesses of mother**				
No	1,43,464 (28.6)	91,962 (30.4)	51,502 (25.7)	<0.001
Yes	3,58,947 (71.4)	2,10,382 (69.6)	1,48,565 (74.3)	
**Illnesses of siblings**				
No	2,80,218 (55.8)	1,83,165 (60.6)	97,053 (48.5)	<0.001
Yes	2,22,193 (44.2)	1,19,179 (39.4)	1,03,014 (51.5)	

# 1 pound was worth about $1.529 in December 2010.

CCI, Charlson comorbidity index; BMI, body mass index; WHR, waist-hip ratio; TDI, Townsend deprivation index; IPAQ, International Physical Activity Questionnaire.

The distributions of all covariates are statistically different between the different CCI groups (Table 1). Age and TDI are probably higher in the CCI > 0 groups than in the CCI = 0 groups, and BMI and WHR are also, although only slightly higher. Whites are the most represented ethnic group (94.6%). Current smokers account for only 10.6% of the total population (9.1% and 12.9% in the CCI = 0 and CCI > 0 groups, respectively). However, current drinkers occupy over 90% of the whole population (93.3 and 89.8% in the CCI = 0 and CCI > 0 groups, respectively). Regarding relative illnesses, over 70% of fathers or mothers suffer from a significant illness, but only 44.2% of siblings have some severe illness (39.4 and 51.5% in the CCI = 0 and CCI > 0 groups, respectively).

We perform univariate logistic regression analyzes on all covariates, and the results indicate that they are all possible factors influencing CCI. After fitting these data into a multivariate logistic regression model, the age, BMI, WHR, TDI, smoking (both previous and current smoking), previous alcohol, maternal smoking around birth, and illnesses of kinship (illnesses of father, mother, or sibling) might be risk factors for CCI. WHR [Odds Ratios (OR) = 11.45, 95%CI = (10.605, 12.363)], previous smoking [OR = 1.226, 95%CI = (1.210, 1.243)], current smoking [OR = 1.662, 95%CI = (1.629, 1.697)], previous alcohol consumption [OR = 1.208, 95%CI = (1.158, 1.261)] and illnesses of sibling [OR = 1.195, 95%CI = (1.180, 1.210)] showed higher values for OR (Table 2). The study also found that non-Asian or Asian British, higher income, moderate or high physical activity, and current alcohol may be protective factors for CCI, and that high income, white [OR = 0.818, 95% CI = (0.785, 0.853)] and current alcohol [OR = 0.798, 95% CI = (0.774, 0.822)] exhibited lower OR (Table 2). We report the AUC and ROC plots for the 10-fold cross-validation of the multivariate logistic regression model in Supplementary Figure 1.

**Table 2:** Univariate and multivariate logistic regression analyzes of factors influencing CCI in all participants [Median (Q1, Q3)/*n* (%)].

**Variables**	**Univariate logistic regression**		**Multivariate logistic regression**	
	***P*** **-value**	**OR (95%CI)**	***P*** **-value**	**OR (95%CI)**
Age (years)	<0.001	1.073 (1.072, 1.074)	<0.001	1.062 (1.061, 1.063)
**Ethnic (Reference: Asian or Asian British)**				
Black or Black British	0.027	0.937 (0.885, 0.993)	<0.001	0.900 (0.846, 0.958)
Mixed	<0.001	0.789 (0.726, 0.857)	0.012	0.891 (0.814, 0.975)
Others	<0.001	0.854 (0.796, 0.916)	<0.001	0.834 (0.774, 0.899)
White	<0.001	0.893 (0.860, 0.927)	<0.001	0.818 (0.785, 0.853)
BMI (Kg/m^2^)	<0.001	1.071 (1.070, 1.073)	<0.001	1.042 (1.040, 1.043)
WHR	<0.001	82.419 (77.176, 88.024)	<0.001	11.45 (10.605, 12.363)
**Income (Reference:**<**18,000 £)**^#^				
18,000–31,000	<0.001	0.654 (0.644, 0.665)	<0.001	0.791 (0.778, 0.805)
31,000–52,000	<0.001	0.461 (0.454, 0.469)	<0.001	0.698 (0.686, 0.711)
52,000–1,00,000	<0.001	0.347 (0.341, 0.354)	<0.001	0.621 (0.609, 0.634)
>1,00,000	<0.001	0.282 (0.273, 0.290)	<0.001	0.534 (0.517, 0.551)
TDI	<0.001	1.047 (1.046, 1.049)	<0.001	1.026 (1.024, 1.028)
**IPAQ (Reference: Low)**				
Moderate	<0.001	0.805 (0.792, 0.818)	<0.001	0.852 (0.838, 0.866)
High	<0.001	0.768 (0.756, 0.780)	<0.001	0.830 (0.816, 0.844)
**Smoking (Reference: No)**				
Previous	<0.001	1.526 (1.508, 1.545)	<0.001	1.226 (1.210, 1.243)
Current	<0.001	1.745 (1.713, 1.778)	<0.001	1.662 (1.629, 1.697)
**Alcohol (Reference: No)**				
Previous	<0.001	1.349 (1.297, 1.403)	<0.001	1.208 (1.158, 1.261)
Current	<0.001	0.717 (0.698, 0.737)	<0.001	0.798 (0.774, 0.822)
Maternal smoking around birth (Reference: No)	<0.001	1.126 (1.112, 1.140)	<0.001	1.103 (1.088, 1.118)
Illnesses of father (Reference: No)	<0.001	1.208 (1.192, 1.225)	<0.001	1.070 (1.055, 1.086)
Illnesses of mother (Reference: No)	<0.001	1.261 (1.245, 1.277)	<0.001	1.089 (1.074, 1.104)
Illnesses of siblings (Reference: No)	<0.001	1.631 (1.613, 1.650)	<0.001	1.195 (1.180, 1.210)

# 1 pound was worth about $1.529 in December 2010.

OR, odds ratios; CI, confidence interval; BMI, body mass index; WHR, waist-hip ratio; TDI, Townsend deprivation index; IPAQ, International Physical Activity Questionnaire.

The rate of CCI > 0 is higher among males (43.3%) than females (36.9%), and the proportion of males with high income, smoking and drinking, and high physical activity is also higher than that of females. Compared to the males, the females have a higher proportion of illnesses of kinship and lower BMI, WHR, and TDI (Table 3).

**Table 3:** Differences in characteristics between males and females [Median (Q1, Q3)/*n* (%)].

**Variables**	**Total** **(*n* = 5,02,411)**	**Female** **(*n* = 2,73,325)**	**Male** **(*n* = 2,29,086)**	***P*** **-value**
Age (years)	58 (50, 63)	57 (50, 63)	58 (50, 64)	<0.001
**Ethnic**				
Asian or Asian British	11,553 (2.3)	5,615 (2.1)	5,938 (2.6)	<0.001
Black or Black British	8,131 (1.6)	4,688 (1.7)	3,443 (1.5)	
Mixed	2,969 (0.6)	1,858 (0.7)	1,111 (0.5)	
Others	4,596 (0.9)	2,611 (1)	1,985 (0.9)	
White	4,75,162 (94.6)	2,58,553 (94.6)	2,16,609 (94.6)	
BMI (Kg/m^2^)	26.75 (24.14, 29.91)	26.13 (23.46, 29.74)	27.31 (24.99, 30.07)	<0.001
WHR, median (Q1, Q3)	0.87 (0.8, 0.94)	0.81 (0.77, 0.86)	0.93 (0.89, 0.98)	<0.001
**Income (£)** ^#^				
< 18,000	1,18,903 (23.7)	69,990 (25.6)	48,913 (21.4)	<0.001
18,000–31,000	1,28,975 (25.7)	72,615 (26.6)	56,360 (24.6)	
31,000–52,000	1,29,124 (25.7)	68,365 (25)	60,759 (26.5)	
52,000–1,00,000	99,117 (19.7)	49,597 (18.1)	49,520 (21.6)	
>1,00,000	26,292 (5.2)	12,758 (4.7)	13,534 (5.9)	
TDI	−2.14 (−3.64, 0.55)	−2.14 (−3.63, 0.49)	−2.12 (−3.65, 0.63)	<0.001
**IPAQ**				
Low	95,267 (19)	51,123 (18.7)	44,144 (19.3)	<0.001
Moderate	2,05,023 (40.8)	1,17,051 (42.8)	87,972 (38.4)	
High	2,02,121 (40.2)	1,05,151 (38.5)	96,970 (42.3)	
**Smoking**				
Never	2,75,033 (54.7)	1,62,915 (59.6)	1,12,118 (48.9)	<0.001
Previous	1,74,038 (34.6)	85,897 (31.4)	88,141 (38.5)	
Current	53,340 (10.6)	24,513 (9)	28,827 (12.6)	
**Alcohol**				
Never	22,492 (4.5)	16,038 (5.9)	6,454 (2.8)	<0.001
Previous	18,174 (3.6)	10,018 (3.7)	8,156 (3.6)	
Current	4,61,745 (91.9)	2,47,269 (90.5)	2,14,476 (93.6)	
**Maternal smoking around birth**				
No	3,54,668 (70.6)	1,95,176 (71.4)	1,59,492 (69.6)	<0.001
Yes	1,47,743 (29.4)	78,149 (28.6)	69,594 (30.4)	
**Illnesses of father**				
No	1,16,383 (23.2)	61,134 (22.4)	55,249 (24.1)	<0.001
Yes	3,86,028 (76.8)	2,12,191 (77.6)	1,73,837 (75.9)	
**Illnesses of mother**				
No	1,43,464 (28.6)	71,362 (26.1)	72,102 (31.5)	<0.001
Yes	3,58,947 (71.4)	2,01,963 (73.9)	1,56,984 (68.5)	
**Illnesses of siblings**				
No	2,80,218 (55.8)	1,47,538 (54)	1,32,680 (57.9)	<0.001
Yes	2,22,193 (44.2)	1,25,787 (46)	96,406 (42.1)	

# 1 pound was worth about $1.529 in December 2010.

CCI, Charlson comorbidity index; BMI, body mass index; WHR, waist-hip ratio; TDI, Townsend deprivation index; IPAQ, International Physical Activity Questionnaire.

Multivariate logistic regression analyzes (univariate logistic regressions were shown in Supplementary Table 2) for females and males separately reveal that mixed-race in female [OR = 0.924, 95% CI = (0.823, 1.038)] and black or black British in male [OR = 0.943, 95% CI = (0.859, 1.035)] are not statistically significant predictors compared with Asian or Asian British (Table 4). High WHR increases the risk of CCI in both males [OR = 19.439, 95% CI = (16.261, 23.241)] and females [OR = 12.575, 95% CI = (11.005, 14.370)], and the effect of WHR on CCI is greater in males (Table 4). The AUC and ROC plots for the 10-fold cross-validation of the multivariate logistic regression model are also presented in Supplementary Figure 1.

**Table 4:** Multivariate logistic regression analyzes of factors influencing CCI in females and males [Median (Q1, Q3)/*n* (%)].

**Variables**	**Females**		**Males**	
	***P*** **-value**	**OR (95%CI)**	***P*** **-value**	**OR (95%CI)**
Age (years)	<0.001	1.051 (1.050, 1.052)	<0.001	1.075 (1.074, 1.076)
**Ethnic (Reference: Asian or Asian British)**				
Black or Black British	0.003	0.880 (0.809, 0.958)	0.216	0.943 (0.859, 1.035)
Mixed	0.184	0.924 (0.823, 1.038)	0.017	0.839 (0.726, 0.969)
Others	0.004	0.863 (0.780, 0.955)	<0.001	0.809 (0.723, 0.904)
White	<0.001	0.842 (0.793, 0.894)	<0.001	0.806 (0.760, 0.855)
BMI (Kg/m^2^)	<0.001	1.043 (1.041, 1.044)	<0.001	1.037 (1.034, 1.039)
WHR	<0.001	12.575 (11.005, 14.370)	<0.001	19.439 (16.261, 23.241)
**Income (Reference:**<**18,000 £)** ^#^				
18,000–31,000	<0.001	0.812 (0.794, 0.830)	<0.001	0.754 (0.734, 0.774)
31,000–52,000	<0.001	0.718 (0.701, 0.735)	<0.001	0.666 (0.648, 0.684)
52,000–1,00,000	<0.001	0.654 (0.636, 0.672)	<0.001	0.586 (0.569, 0.604)
>1,00,000	<0.001	0.541 (0.516, 0.567)	<0.001	0.525 (0.502, 0.549)
TDI	<0.001	1.023 (1.020, 1.026)	<0.001	1.029 (1.026, 1.032)
**IPAQ (Reference: Low)**				
Moderate	<0.001	0.839 (0.820, 0.858)	<0.001	0.871 (0.849, 0.893)
High	<0.001	0.838 (0.818, 0.857)	<0.001	0.836 (0.816, 0.857)
**Smoking (Reference: No)**				
Previous	<0.001	1.186 (1.164, 1.208)	<0.001	1.248 (1.224, 1.273)
Current	<0.001	1.658 (1.610, 1.708)	<0.001	1.668 (1.621, 1.717)
**Alcohol (Reference: No)**				
Previous	<0.001	1.223 (1.158, 1.291)	<0.001	1.185 (1.102, 1.275)
Current	<0.001	0.793 (0.765, 0.822)	<0.001	0.798 (0.754, 0.844)
Maternal smoking around birth (Reference: No)	<0.001	1.098 (1.079, 1.119)	<0.001	1.108 (1.086, 1.130)
Illnesses of father (Reference: No)	<0.001	1.054 (1.033, 1.075)	<0.001	1.086 (1.063, 1.109)
Illnesses of mother (Reference: No)	<0.001	1.076 (1.055, 1.097)	<0.001	1.097 (1.075, 1.119)
Illnesses of siblings (Reference: No)	<0.001	1.169 (1.149, 1.189)	<0.001	1.224 (1.202, 1.247)

# 1 pound was worth about $1.529 in December 2010.

OR, odds ratios; CI, confidence interval; CCI, Charlson comorbidity index; BMI, body mass index; WHR, waist-hip ratio; TDI, Townsend deprivation index; IPAQ, International Physical Activity Questionnaire.

**Figure 2:**
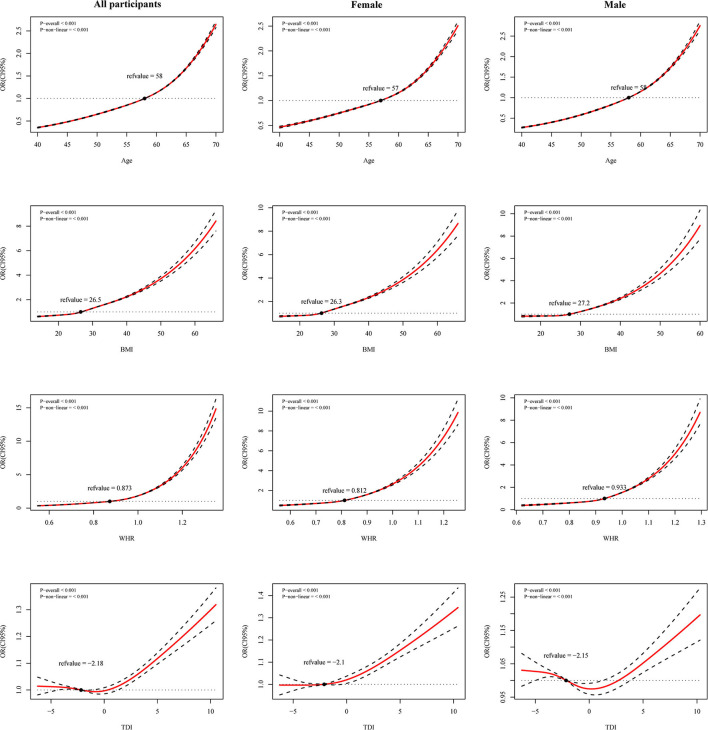
Restricted cubic spline (RCS) curves for age, Townsend deprivation index (TDI), waist-hip ratio (WHR) and body mass index (BMI) with Charlson comorbidity index (CCI) risk in all participants, female and male. Adjusted for ethnicity, smoking, alcohol, income, and maternal smoking at birth.

We fit restricted cubic splines with four sections in a logistic regression model to investigate the potential non-linear association of age, TDI, WHR, and BMI with the risk of CCI. Associations between age, BMI, WHR, and CCI risk are J-shaped for all participants, males, and females. The elevated risk is minimal for low age, BMI, and WHR (Figure 2) but increased markedly above a certain value. We found a U-shaped association between TDI and CCI risk for all participants. The risk is flat or decreased slightly at lower TDIs and begins to increase at a faster rate at TDI above 0. Similar trends are found in males, but with more magnitude. It fell rapidly at TDI = −3 and rose at around TDI = 0.5 (Figure 2). The lowest point of CCI risk is estimated at a TDI of −0.5 and 0.5 for the whole population and men, respectively (Figure 2).

In the sensitivity analysis, 88,928 males and 1,55,902 females, excluding individuals with missing values from the analysis does not change our main results. CCI risks show the same relationship except for ethnicity in females and mixed ethnicities in males. We plotted forest plots to visualize the comparison between the primary outcome and the sensitivity analysis (Supplementary Figures 2–4).

## 4. Discussion

In this large study of cross-sectional data, we investigate the factors influencing CCI in a UK biobank of over 5,00,000 participants. We represent different levels of disease by varying CCI scores and report the results of factors that might influence the risk of CCI in all participants, males, and females. Our study differed from others in that we further analyze the possible non-linear relationships between age, BMI, WHR, TDI, and CCI risk. There are several critical points that we could take away from this research:

1) Increasing age and smoking (both previous and current) seem to be risk factors for reduced health, and moderate and vigorous physical activity seem to be beneficial to health.
2) Different drinking statuses may have opposite effects on CCI risk. Current alcohol consumption could be a protective factor for CCI risk. It may take years to see the negative impacts of current alcohol consumption.
3) High WHR and high BMI are significant predictors of CCI risk, with WHR having a greater effect.
4) TDI and CCI risk is U-shaped and sex-specific, implying that the impact of socioeconomic factors on CCI is more complex.

### 4.1. Age, ethnicity and illness of relatives

In this analysis, we do not use age to calculate the CCI score (25) but rather as an independent variable in the regression model to explore its non-linear relationship with CCI risk. It found that the curve inflected at around age 60 and that the OR increased rapidly. Such a feature was in line with the current principles of CCI assignment (25). Some multimorbidities, such as cardiovascular disease, hypertension, and diabetes had previously been higher in South Asians (26). Our results also demonstrate that the ethnic category “Asian or Asian British” might have a negative effect on CCI risk. This might be attributed to the fact that race could influence individual health through cultural habits, behavioral patterns, etc. Besides, genetics and susceptibility to infections might also be important in accounting for health differences across races (27); after all, some infections causing respiratory diseases were also an essential component of CCI. The presence of relatives with more severe diseases could also be a risk factor for CCI, especially in those whose siblings suffered from the illness. After identifying non-modifiable predisposing risk factors, the focus should be modifiable behavioral patterns.

### 4.2. Smoking, alcohol, and physical activity

Smoking and poor physical activity were considered harmful to health (28–30), and our results provide further evidence. Previous alcohol is a risk factor for health, whereas current alcohol is the opposite. Previous drinkers often abstained from alcohol because of poor health (31, 32) and showed that previous drinkers were associated with an increased risk of CCI. Some researchers have found that the association between alcohol intake and cardiovascular disease incidence was often reported as a j-shaped curve (33). Moderate alcohol consumption was not only linked to a reduced relative risk of cardiovascular disease but was also beneficial for type 2 diabetes (34). Apart from the amount of alcohol, genetic factors and frequency of drinking contributed significantly to differences in alcohol metabolism. Therefore, current drinking should be cautiously treated due to CCI protective factors.

### 4.3. WHR and BMI

In contrast to BMI, WHR, an important indicator for measuring obesity, has rarely been employed to investigate its impact on human health. An NHANES (National Health and Nutrition Examination Survey) study first demonstrated that obesity, determined by WHR, was associated with an increased risk of cardiovascular mortality (35). A meta-analysis showed that WHR was a valid predictor of heart attack risk with strong predictive power (36), consistent with our findings. High WHR and high BMI increased the risk of CCI in both sex but shows substantial differences in the magnitude of the effect. These differences may be because two different obesity characteristics affect two different diseases (37) (WHR was highly correlated with visceral fat, whereas BMI was highly correlated with subcutaneous fat). Previous studies have also found inconsistencies between WHR and BMI in predicting some outcome events (38). In addition, guideline developers did not consider individuals with high WHR as a priority population for prevention programs (39). This suggests that we must focus our lifestyle changes and other prevention strategies on people with central obesity characterized by high WHR.

### 4.4. TDI

The TDI was calculated by combining various factors, including employment and housing, and to some extent, indicates people's behavioral patterns (40). Using the TDI to represent socioeconomic status allowed the underestimation caused by a single indicator to be avoided. Our findings suggest that high deprivation indices increase the risk of CCI, which is consistent with the results of previous studies. Higher deprivation levels were associated with an increased risk of multiple morbidities (5, 41, 42) and poor health status (43). A survival analysis of cardiovascular disease showed that individuals in geographic areas with higher socioeconomic disadvantage have higher mortality rates over a follow-up period of more than 10 years, after controlling for multiple variables (44). A study by Riley et al. (45) also concluded that the risk of diabetes-related foot disease increased with increasing indices of deprivation. Cardiovascular disease and diabetes and its complications were both important components of CCI. The association between high levels of deprivation and poor health outcomes might be related to poorer collective resources in deprived areas (46). In addition, poor self-management and unhealthy lifestyle factors (e.g., long screen use time, irregular diet) due to poverty had a higher impact on health and mortality (47, 48). These might explain our study's association between deprivation levels and CCI risk.

In the present study, we found a U-shaped association between TDI and CCI risk among all participants. Foster's study revealed a lower risk of all-cause mortality, cardiovascular mortality, and cardiovascular morbidity in the moderately healthy lifestyle population compared to the least deprived and most deprived populations (47). Which was similar to our findings. However, this U-shaped association differed in the subsequent sex analysis. For the TDI-CCI risk association, we only observe a U-shape in the males, while a J-shape in the females. Interestingly, males with a low social deprivation index alone are associated with a high risk of CCI. This may reflect that the effect of socioeconomic status on CCI risk is more complex in males, indicating sex differences in the effect of economic status on health. This situation could be related to the differences in employment status and income between males and females. A Spanish study reported that women were more likely to experience poor health, employment conditions, and lower earnings when employed compared to men (49). White et al. (50) conducted a study exploring health factors from a male perspective, which showed significant sex differences in several social factors that harm health.

It could be noticed that although absolute inequality was declining in some countries (51), the impact of socioeconomic factors on health remained severe. Deprivation may be an essential modifiable factor in health costs, placing greater demands on public health strategies and the rational allocation of resources.

### 4.5. Limitations

There were some limitations to our study.

1) The cross-sectional study was unable to determine causality, i.e., it was not clear whether health led to behavior change or whether behavior led to different health outcomes;
2) Sampling and questionnaire data were inevitably subject to sampling error, recall bias, reporting bias, and non-response bias;
3) Due to the large sample size, even minimal differences could be statistically significant. Significant associations for some variables might be based on minor absolute differences, such as WHR in the two groups;
4) The sample was mainly from the UK and may lack generalizability to the world population;
5) The independent variables included in the study were limited to general conditions and did not include information such as dietary information and blood tests, which will probably be explored in a future study.

## 5. Conclusions

In summary, increases in WHR, BMI, and TDI are significant predictors of poor health, and WHR shows a greater role. The impact of deprivation indices on health showed differences by sex. Socioeconomic factors, such as income and TDI, are associated with CCI. The association of social status differences caused by these socioeconomic factors with health conditions should be considered. Our research reveals several demographic and behavioral factors that might be associated with health conditions. This association allows individuals to estimate their health conditions based on their characteristics and to increase awareness of disease prevention and harm reduction due to disease. This could have important implications for disease prevention and health improvement. Factors might interact with each other. Therefore, a comprehensive, rational, and robust intervention will be necessary for health. Given the progressive increase in the number and prevalence of chronic diseases in individuals in recent years and our results, it remained crucial to continue investigating other factors that could be relevant to CCI.

## Data availability statement

Publicly available datasets were analyzed in this study. This data can be found here: https://www.ukbiobank.ac.uk/.

## Author contributions

DM developed the study design. CW, XZ, and BL planned the analyzes and conducted the data analysis. All authors participated in result interpretation, revised all contents of the manuscript, and critically reviewed and approved the submitted manuscript.
