# Supplementary material for: A study of factors impacting disease based on the Charlson Comorbidity Index in UK Biobank
Source: Front Public Health. 2023 Jan 9;10:1050129. doi: 10.3389/fpubh.2022.1050129 (PMC9868818; doi:10.3389/fpubh.2022.1050129)
Supplement: Supplementary file 1 [file Table_1.DOCX]

**Table S1 The score of diseases.**

| Num | Diseases | CCI Score |
| --- | --- | --- |
| 1 | Myocardial infarction | 1 |
| 2 | Congestive heart failure | 1 |
| 3 | Peripheral vascular disease | 1 |
| 4 | Cerebrovascular disease | 1 |
| 5 | Dementia | 1 |
| 6 | Chronic pulmonary disease | 1 |
| 7 | Rheumatic disease | 1 |
| 8 | Peptic ulcer disease | 1 |
| 9 | Diabetes without chronic complication | 1 |
| 10 | Diabetes with chronic complication | 2 |
| 11 | Moderate or severe liver disease | 3 |
| 12 | Renal disease | 2 |
| 13 | Hemiplegia or paraplegia | 2 |
| 14 | Any malignancy, including lymphoma and leukemia, except malignant neoplasm of skin | 2 |
| 15 | Metastatic solid tumor | 6 |
| 16 | Mild liver disease | 1 |
| 17 | AIDS HIV | 6 |

**Table S2 Univariate logistic regression analyses of factors influencing CCI in females and males [Median (Q1, Q3)/n (%)].**

| Variables | Females | |  | Males | |
| --- | --- | --- | --- | --- | --- |
|  | *P* | OR (95%CI) |  | *P* | OR (95%CI) |
| **Age (year)** | <0.001 | 1.062(1.061,1.063) |  | <0.001 | 1.085(1.084,1.086) |
| **Ethnic (Reference: Asian or Asian British)** | |  |  |  |  |
| Black or Black British | 0.259 | 1.047(0.967,1.133) |  | <0.001 | 0.850(0.781,0.926) |
| Mixed | 0.013 | 0.871(0.781,0.971) |  | <0.001 | 0.733(0.642,0.836) |
| Others | 0.086 | 0.920(0.836,1.012) |  | <0.001 | 0.812(0.733,0.901) |
| White | <0.001 | 0.890(0.844,0.940) |  | 0.002 | 0.922(0.875,0.971) |
| **BMI (Kg/m^2^)** | <0.001 | 1.070(1.068,1.072) |  | <0.001 | 1.069(1.067,1.071) |
| **WHR** | <0.001 | 252.327(224.931,283.1) |  | <0.001 | 782.367(683.18,896.167) |
| **Income (Reference: Less than 18,000)** | |  |  |  |  |
| 18,000 to 31,000 | <0.001 | 0.666(0.652,0.680) |  | <0.001 | 0.619(0.604,0.634) |
| 31,000 to 52,000 | <0.001 | 0.475(0.465,0.486) |  | <0.001 | 0.418(0.408,0.429) |
| 52,000 to 100,000 | <0.001 | 0.362(0.354,0.372) |  | <0.001 | 0.306(0.298,0.314) |
| Greater than 100,000 | <0.001 | 0.275(0.263,0.288) |  | <0.001 | 0.259(0.249,0.270) |
| **TDI** | <0.001 | 1.050(1.048,1.053) |  | <0.001 | 1.044(1.041,1.046) |
| **IPAQ (Reference: Low)** |  |  |  |  |  |
| Moderate | <0.001 | 0.785(0.769,0.802) |  | <0.001 | 0.845(0.825,0.864) |
| High | <0.001 | 0.76(0.744,0.777) |  | <0.001 | 0.768(0.750,0.785) |
| **Smoking (Reference: No)** |  |  |  |  |  |
| Previous | <0.001 | 1.319(1.297,1.342) |  | <0.001 | 1.714(1.684,1.745) |
| Current | <0.001 | 1.674(1.629,1.720) |  | <0.001 | 1.758(1.712,1.804) |
| **Alcohol (Reference: No)** |  |  |  |  |  |
| Previous | <0.001 | 1.260(1.199,1.325) |  | <0.001 | 1.415(1.325,1.511) |
| Current | <0.001 | 0.644(0.624,0.665) |  | <0.001 | 0.777(0.740,0.817) |
| **Maternal smoking around birth (Reference: No)** | <0.001 | 1.126(1.107,1.146) |  | <0.001 | 1.113(1.093,1.133) |
| **Illnesses of father (Reference: No)** | <0.001 | 1.189(1.167,1.212) |  | <0.001 | 1.248(1.224,1.272) |
| **Illnesses of mother (Reference: No)** | <0.001 | 1.267(1.244,1.290) |  | <0.001 | 1.305(1.282,1.329) |
| **Illnesses of siblings (Reference: No)** | <0.001 | 1.579(1.555,1.604) |  | <0.001 | 1.746(1.717,1.775) |
